# Long-term impact of sagittal malalignment on hardware after posterior fixation of the thoracolumbar spine: a retrospective study

**DOI:** 10.1186/s12891-020-03405-z

**Published:** 2020-06-16

**Authors:** Mahmoud Elshamly, Reinhard Windhager, Stefan Toegel, Josef Georg Grohs

**Affiliations:** 1 Department of Orthopedics and Trauma Surgery, Division of orthopedics, Medical University of Vienna, Waehringer Guertel 18-20, A-1090 Vienna, Austria; 2 Karl Chiari Lab for Orthopedic Biology, Department of Orthopedics and Trauma Surgery, Medical University of Vienna, Waehringer Guertel 18-20, A-1090 Vienna, Austria; 3 Ludwig Boltzmann Cluster for Arthritis and Rehabilitation, Vienna, Austria

**Keywords:** Sagittal alignment, Posterior fixation, Failure of the implants, Thoracolumbar spine

## Abstract

**Background:**

The importance of sagittal alignment in healthy individuals and in reconstructive spinal surgery has been studied over the last 15 years. The aim of the present study was to assess the long-term effects of abnormal sagittal alignment on hardware after posterior thoracolumbar spinal fusion.

**Methods:**

Patients who had undergone revision surgery (revision cohort, *n* = 34) due to breakage of their implants were compared retrospectively with patients who had intact implants at the final follow-up investigation after a long posterior thoracolumbar and/or lumbar spinal fusion (control cohort, *n* = 22). Clinical data and radiological parameters including the sagittal vertical axis (SVA), pelvic incidence (PI), lordosis gap (LG), pelvic tilt (PT), sacral slope (SS), lumbar lordosis (LL), thoracic kyphosis (TK), and the femoral obliquity angle (FOA) were assessed on full-spine lateral radiographs obtained in regular standing position. Data were analysed using descriptive statistics, parametric and non-parametric inferential statistics.

**Results:**

Patients in the breakage group (female *n* = 21, male *n* = 9, mean age 60.9 ± 15.6 years) had a higher anterior shift of the C7 plumb line (SVA) (*p* = 0.02), retroversion of the pelvis (PT) (*p* < 0.001), PI-LL mismatch (LG) (*p* = 0.001), and PI (*p* = 0.002) than the intact group (female *n* = 10, male *n* = 12, mean age 65.7 ± 12.4 years). No significant difference was registered between groups in regard of SS, LL, TK, FOA, and the mean number of comorbidities**.**

**Conclusion:**

Failure of restoration of the SVA and the LG to the acceptable ranges, especially in patients with a high PI, may be regarded as a risk factor for the long-term failure of implants after posterior thoracolumbar spinal fusion.

## Background

The importance of sagittal alignment in healthy individuals and in reconstructive spinal surgery has been studied over the last 15 years [1–5]. Indeed, the conservation of sagittal alignment is a crucial factor for the ability of the human body to maintain a standing position with minimal energy expenditure [6]. Clinically, the assessment of sagittal alignment is a mandatory aspect of preoperative planning in order to minimize postoperative complications, including adjacent segment disease and progressive deformity [5, 7].

Le Huec et al. reported a significant correlation between a high postoperative PT on the one hand, and postoperative back pain and the risk of adjacent segment degeneration on the other [8].

A high PI and failure to restore a reasonable lumbar lordosis were found to be risk factors for the development of degenerative spondylolisthesis and the failure of lumbopelvic fixation after long fusions in patients with adult spinal deformities (ASD) [9].

Berjano et al. described an abnormal SVA as a risk factor for the failure of surgery and revision surgery after spinal fusion [10]. Yamada et al. registered an association between the patients’ satisfaction after corrective spinal fusion surgery and their SVA at the final follow-up [11]. Furthermore, the PI-LL mismatch (LG) after long-segment fusion surgery, particularly for ASD, was found to be associated with residual postoperative symptoms such as low back pain (LBP), lower extremity pain, and numbness [12]. Numerous studies demonstrated a strong correlation between the improvement of sagittal deviation and clinical outcomes after instrumented fusion for various degenerative lumbar spine diseases [11–17].

The evaluation of sagittal spinal alignment involves the assessment of various parameters such as pelvic incidence (PI), lumbar lordosis (LL), pelvic tilt (PT), sacral slope (SS), lordosis gap (LG), thoracic kyphosis (TK), sagittal vertical axis (SVA), and the femoral obliquity angle (FOA) [1, 18–22].

Despite numerous investigations of the effect of sagittal alignment on the clinical outcome of spinal fusion surgery, we lack data concerning the role of sagittal malalignment in the failure of hardware after posterior fixation of the thoracolumbar spine. We hypothesized an association between sagittal malalignment, metal fatigue, and subsequent failure of implants after posterior thoracolumbar spine fusion.

## Methods

Two cohorts of patients who underwent surgery at one institution were reviewed retrospectively. The investigation was approved by the local ethics committee (approval number 1156/2017).

The revision cohort consisted of all patients who underwent revision surgery due to breakage of the implanted rods and/or screws between 2010 and 2016. The control cohort consisted of consecutive patients who had undergone a fusion at a minimum of three levels in the lumbar and/or thoracolumbar spine, had intact implants at the final follow-up visit, and provided their informed consent to participate in the study. Clinical results in controls were assessed with the Oswestry disability index and the visual analogue scale (VAS).

Patients with concomitant neuromuscular, psychological, or malignant disease, traumatic spinal lesions, infectious spinal disease, or Scheuermann’s kyphosis [23] as the indication for surgery, fusion systems other than rods and screws, and patients with a coronal plumb line exceeding 4 cm were excluded from the study, as the mechanical overload in the coronal plane is independent of sagittal alignment. The aim of the study was to evaluate the effect of sagittal malalignment on the durability of the implants.

Clinical, surgical, and radiographic data obtained from the hospital records were assessed. Standard anteroposterior and lateral radiographs of the full spine in standing position were analysed for each patient.

In the revision cohort, we assessed the preoperative radiographs taken before the index surgery and the radiographs obtained at the time of the patients’ presentation in order to define the cause of the symptoms and prepare for potential revision surgery. In the control cohort, the radiographs were obtained at the final follow-up visit.

The following parameters were determined on lateral radiography for each patient: pelvic incidence (PI), lumbar lordosis (LL), pelvic tilt (PT), sacral slope (SS), lordosis gap (LG) or PI-LL mismatch, thoracic kyphosis (TK), sagittal vertical axis (SVA), and the femoral obliquity angle (FOA) [1, 18–22].

In controls, clinical outcomes were assessed at the final follow-up using the ODI and the VAS score for leg and back pain [24].

### Statistical analysis

The SPSS software (IBM SPSS Statistics Version 23) was used for statistical analysis. An unpaired t-test was employed to evaluate mean values of normally distributed data (demographic data, LL, SS, PT, PI, SVA, TK and LG), while the Mann-Whitney U test was used to assess mean values of not normally distributed data (FOA). Pearson’s correlation analysis was employed to assess the relationship between radiological parameters and the VAS and ODI scores in the control cohort. A multivariate binary logistic regression analysis (Wald test) was performed to define radiological parameters that could be a risk factor for the breakage of implants.

## Results

Demographics and patients’ characteristics are summarized in Table 1.

**Table 1:** Demographics and patient characteristics

	Revision cohort mean ± SD	Control cohort mean ± SD	*p*-values
Number F/M	(*n* = 30) M = 9 F = 21	(*n* = 22) M = 12 F = 10	
Age (years)	62.5 ± 15.7	65.7 ± 12.4	0.23
BMI	26.9 ± 6.4	26.8 ± 4.7	0.66
Number of fixed segments	7.6 ± 3.6	5.6 ± 2.4	0.21
Follow-up (years)	3.6 ± 2.9	3.8 ± 1.7	0.9

An independent sample t-test revealed no significant difference between the two cohorts in terms of age, BMI, the number of fixed segments, and the duration of follow-up

A review of the patients’ medical history revealed that 30 patients in the revision cohort and 18 patients in the control group had various cardiovascular, respiratory, endocrinal, neurological, osteoporotic, and gastrointestinal diseases. The mean number of comorbidities was 2.4 ± 1.8 and 2.3 ± 1.9 in the two groups, respectively (*p* = 0.9). In the revision cohort, three patients had ASA1, 17 had ASA2, nine had ASA3, and one patient had ASA4. In the control cohort, three patients had ASA1, 13 had ASA2, four had ASA3, and two patients had ASA4.

The duration of follow-up did not differ significantly between groups (*p* = 0.9).

In the revision cohort, 11 patients presented to the department after failure of the index operation performed at other hospitals, while 19 patients were operated on at our department by two of our surgeons.

The indication for the index operation was correction of scoliosis in 11 patients, while the indication in the remaining patients was spondylochondrosis [25] and instability. The operative techniques used were postero-lateral fusion (PLF) with or without transforaminal lumbar interbody fusion (TLIF), Smith-Peterson osteotomy (SPO), or combinations of the two techniques. No pedicle subtraction osteotomy (PSO) was performed in this group. The lower instrumented segment was L5 in five patients and S1 in the remaining patients. The upper instrumented segments varied from L3 to Th11.

Sagittal parameters measured on full-spine radiographs before the index operation revealed sagittal malalignment that was not corrected by the index operation and did not differ significantly from the sagittal malalignment measured at the time of the patients’ presentation with broken rods and/or screws (*p* > 0.05); the data are shown in Table 2.

**Table 2:** Radiographic measurements in the revision cohort

	First operation	Revision surgery	*p*-values
LL (°) mean ± SD	27.4 ± 15.7	34.6 ± 20.3	0.86
SS (°) mean ± SD	29.9 ± 6.7	34.4 ± 10.2	0.74
PT (°) mean ± SD	32.6 ± 7.7	33.3 ± 9.6	0.95
PI (°) mean ± SD	62.5 ± 10.7	67.7 ± 14.7	0.8
SVA (mm) mean ± SD	104.4 ± 48.5	112.2 ± 64.8	0.83
TK (°) mean ± SD	24 ± 15.3	26.4 ± 16.7	0.61
LG (°) mean ± SD	24.2 ± 16.5	33.9 ± 19.5	0.90
FOA Median / IQ range	10 ± 13	8 ± 12	0.94

The means of each parameter revealed no significant differences between the first operation and the time of revision surgery

Breakage sites of screws and/or rods and sites of screw loosening in the revision cohort are listed in Table 3.

**Table 3:** Sites of screw breakage, rod breakage, and screw loosening

Level of the problem	Number of screw breakages	Number of rod breakages	Number of screw loosenings
Th11		3	
Th12	1	2	
L1		1	
L2	1		
L3	1	3	1
L4	2	2	
L5	4	3	
S1	4	3	5

The control cohort consisted of 22 patients who were operated by two surgeons at the department. Three patients had degenerative scoliosis while the remaining patients had spondylochondrosis and instability. The surgical procedure used was PLF with or without TLIF. PSO at the level of L4 was performed in two patients. The lower instrumented segment was L3 and L5 in two patients, and S1 in the remaining patients. The upper instrumented segments varied from L2 to Th11.

**Fig. 1:**
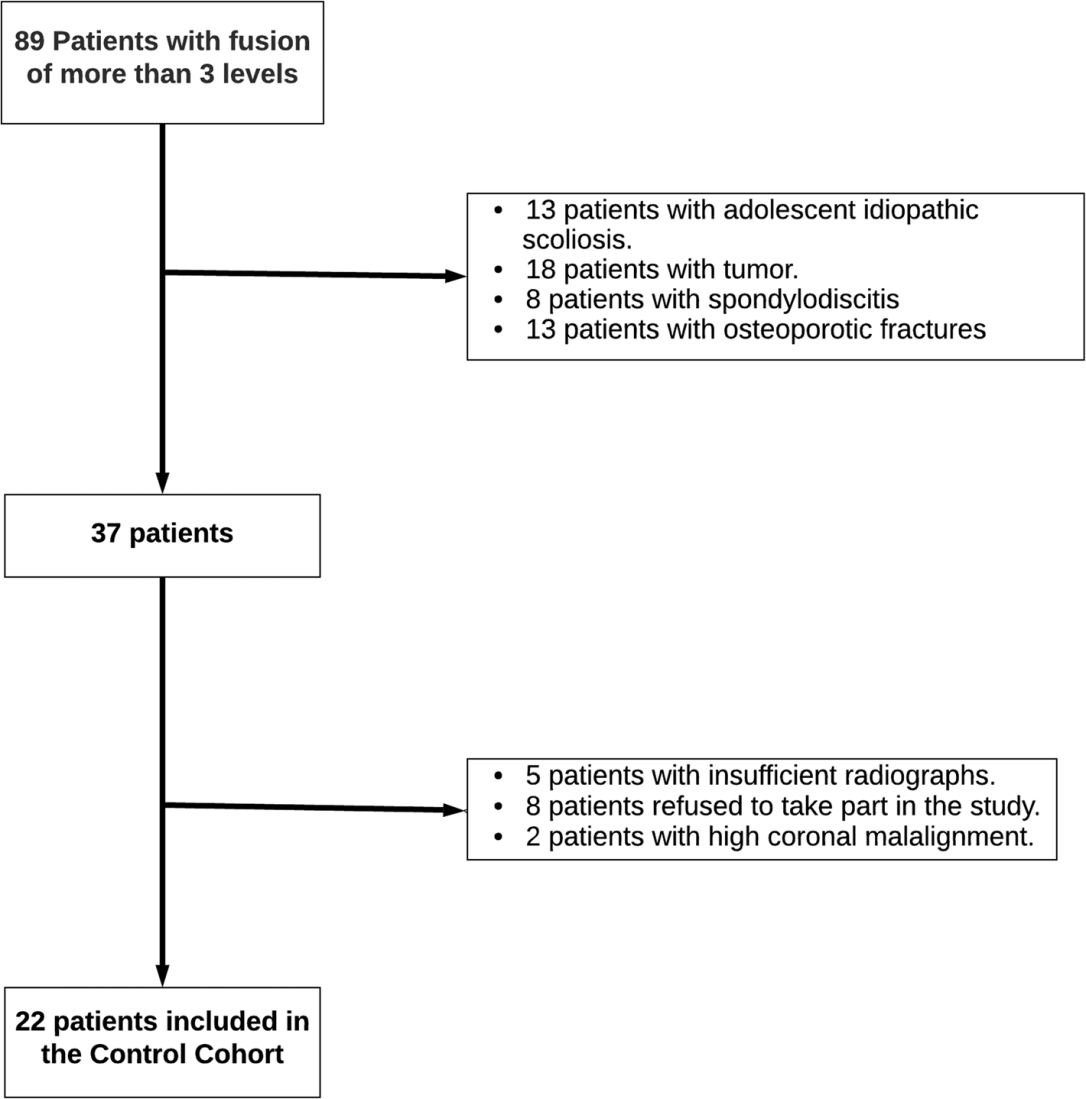
Flowchart of patients who were requested to be part of the control group

Figure 1 shows the number of contacted patients and the numbers excluded from the control group.

Radiological assessment revealed that the anterior shift at C7 was higher in the revision cohort than in controls (mean SVA values (±SD) values 112.2 ± 64.8 vs. 67.1 ± 32.6, respectively, *p* = 0.02). The PI-LL mismatch (LG) was higher in the revision cohort than in controls (mean LG values (±SD) were 33.9 ± 19.5 vs. 11 ± 9.7, respectively, *p* = 0.001). Pelvic retroversion was higher in the revision cohort than in controls (mean PT values (±SD) were 33.3 ± 9.6 vs. 23.5 ± 7.6, respectively, *p* = 0.001). However, there was no significant difference in FOA between the two groups. Pelvic incidence was higher in the revision cohort than in controls (*p* = 0.002). However, no statistically significant differences were noted between groups in regard of their mean values of LL, SS, TK, and the AP C7 plumb line (*p* > 0.05). Radiographic measurements are listed in Table 4.

**Table 4:** Radiographic measurement

	Revision cohort	Control cohort	*p*-values*	*P*-values Wald test
LL (°) mean ± SD	34.6 ± 20.3	43.4 ± 14.3	0.15	0.366
SS (°) mean ± SD	34.4 ± 10.2	30.8 ± 9.5	0.22	0.162
PT (°) mean ± SD	33.3 ± 9.6	23.5 ± 7.6	**0.001**	0.005
PI (°) mean ± SD	67.7 ± 14.7	54.7 ± 12.4	**0.002**	0.006
SVA (mm) mean ± SD	112.2 ± 64.8	67.1 ± 32.6	**0.02**	0.037
TK (°)mean ± SD	26.4 ± 16.7	30.6 ± 14.7	0.60	0.621
LG (°) mean ± SD	33.9 ± 19.5	11 ± 9.7	**0.001**	0.004
FOA Median / IQ range	8 ± 12	6 ± 6	0.7	0.357

*The mean values of PI, PT, SVA, and LG were significantly lower in controls than in the revision cohort (independent sample t-test). A multivariate binary logistic regression analysis (Wald test) showed significant differences in PT, PI, SVA, and LG between the revision cohort and controls

A multivariate binary logistic regression analysis revealed significant differences between the revision group and controls in regard of PT (*p* = 0.005), PI (*p* = 0.006), SVA (*p* = 0.037), and LG (*p* = 0.004); the data are listed in Table 4.

Clinical assessment of controls revealed a moderate correlation between SVA and the total ODI score (*r* = 0.56, *p* = 0.01) as well as the VAS score for back pain (*r* = 0.4, *p* = 0.04). In the revision cohort, patients presented with disabling back pain of sudden onset. Radiographs were obtained to determine the cause of pain, and revision surgery was performed. Under these circumstances, pain and disability were not evaluated before surgery.

## Discussion

Several studies have reported the debilitating course of sagittal malalignment, which requires enormous energy expenditure to maintain the standing position and subsequently impairs the patient’s quality of life [7, 26–28]. However, to the best of our knowledge, this is the first study addressing the effects of postoperative sagittal malalignment on the durability of implants after a long posterior spinal fixation of the lumbar and/or thoracolumbar spine.

Notably, in the present study, the sagittal parameters measured on full-spine radiographs obtained before the index operation revealed sagittal malalignment that was not corrected by the index operation and did not differ significantly from the sagittal malalignment registered when the patients presented with broken rods and/or screws after a mean follow-up period of 3.6 ± 2.9 years. This confirms the presence of sagittal malalignment after the index operation and contradicts the thesis that the broken implants were the reason for sagittal malalignment.

Based on conventional X-rays, Schwab et al. reported the role of PT, PI, and LL combined with SVA in predicting a patient’s disability [29]. The authors proposed threshold values for severe impairment (Oswestry disability index (ODI) > 40), which included PT ≥ 22°, SVA ≥ 47 mm, and PI-LL (LG) ≥ 11° [28]. Moreover, the authors emphasized the significance of restoring a low SVA and PT, and an LL proportional to PI as critical goals when planning realignment surgery for adult spinal deformity [7, 30]. In this context, our control cohort had an abnormally high mean SVA that was manifested clinically as an increase in total ODI and VAS scores. The revision cohort had a much higher SVA compared to controls. Patients in the revision cohort sought medical advice and subsequent surgery because of pain.

In the revision cohort, the second operation was performed on average 3.9 years after the index operation; this interval did not differ from the follow-up period in controls.

PI-LL matching, which was defined in our study as LG, is a vital tool in planning surgery for the correction of sagittal deformity [7], as well as a critical radiological parameter for establishing the clinical outcome [7]. In a prospective multicentre study, Schwab et al. predicted disability (defined as ODI > 40) on the basis of a PI-LL ≥ 11° [28]. However, patients with an abnormal LG and a normal SVA, which is compensated sagittal malalignment, may experience severe disability [31]. Aoki et al. demonstrated the effect of PI-LL mismatch on the persistence of postoperative low back pain, leg pain, and numbness [12]. According to Yamada et al., patients with a postoperative PI-LL mismatch were less satisfied with the surgical outcome [11]. Restoration of lumbar lordosis and correction of PI-LL mismatch prevent sagittal decompensation after long spinal fusion surgery [7]. Failure to restore SVA and LG to the normal range in minimally invasive spinal deformity surgery was associated with poor clinical outcomes [32]. In our study, patients who experienced breakage of their implants had a higher SVA and LG than those without failure of their implants. The higher LG values in the revision cohort indicate that an adequate degree of lordosis was not restored in patients with a higher PI, which may imply that the implants had to sustain higher loads, resulting in metal fatigue.

PT is commonly described as a compensatory mechanism. In the presence of sagittal malalignment, the individual will try to maintain posture by retroversion of the pelvis (an increase of PT); this has a significant impact on the clinical outcome and as well as the patient’s satisfaction [7]. A postoperative PT < 20° is necessary to obtain an optimal result. The realignment of SVA < 50 mm in the presence of a high PT means that the patient is still compensating for a residual structural spinal deformity [7]. Accordingly, several studies reported on the correlation between clinical outcomes and postoperative PT [8, 17, 33–36]. In our investigation, the presence of a high PT in the revision cohort indicated that the patients’ compensatory mechanisms were exhausted. The residual global malalignment was confirmed by a high SVA, which may aggravate stress on the hardware.

PI is a fixed anatomical parameter of primary importance, commonly used to define spinopelvic morphotypes or the required lumbar alignment under optimal conditions [37, 38]. The restoration of a low sagittal vertical axis and pelvic tilt values when planning realignment surgery for adult spinal deformity should be combined with a proportionate lumbar lordosis and pelvic incidence [37–40]. Woojin et al. reported that a large PI was a risk factor for the failure of lumbopelvic fixation after long construct fusion in adults with a spinal deformity [9]. According to Cho et al., the most significant risk factors for sagittal decompensation (of which an SVA falling anteriorly > 8 cm was considered the most important) were preoperative sagittal imbalance and a high PI. In this context, the mean PI of patients in our revision cohort was significantly higher than the mean PI of controls. In contrast to patients with a low PI, compensation is significantly limited in patients with a high PI because of full femur extension; the iliofemoral ligament becomes tight in the anterior aspect, thus preventing full verticalisation of the sacrum.

A number of other risk factors have been reported for the failure of fusion. Cho et al. registered a propensity for breakage of materials in patients with comorbidities and a preoperative coronal malalignment [9]. However, in the present study we registered no significant difference in the mean numbers of comorbidities between the two cohorts, implying that comorbidities did not influence the breakage of implants.

The relatively small size of the sample is a potential limitation of the study. Nevertheless, the statistical analysis revealed significant differences between the two groups in regard of various important radiographic parameters. Further biomechanical studies will have to address the role of a high SVA, LG, and PI as risk factors for the breakage of implants.

## Conclusion

Failure to restore the SVA to the acceptable range and the presence of a high LG, especially in patients with a high PI, may be regarded as risk factors for the long-term failure of implants after a posterior thoracolumbar spine fusion. These risk factors imply that the failure of surgical restoration of sagittal alignment with subsequent debilitation of compensatory mechanisms may increase stress on the hardware, lead to metal fatigue, and aggravate the risk of rod and/or screw breakage. Additional biomechanical studies will have to assess the importance of these factors.

## Data Availability

The datasets used and/or analysed for the current study can be provided by the corresponding author on reasonable request.
